# Maize (*Zea mays L.*) Seedlings Rhizosphere Microbial Community as Responded to Acidic Biochar Amendment Under Saline Conditions

**DOI:** 10.3389/fmicb.2021.789235

**Published:** 2021-12-14

**Authors:** Mukesh Kumar Soothar, Abdoul Kader Mounkaila Hamani, Muhammad Fahad Sardar, Mahendar Kumar Sootahar, Yuanyuan Fu, Riffat Rahim, Jay Kumar Soothar, Saleem Maseeh Bhatti, Sunusi Amin Abubakar, Yang Gao, Jingsheng Sun

**Affiliations:** ^1^Key Laboratory for Crop Water Requirement and Regulation of Ministry of Agriculture and Rural Affairs, Farmland Irrigation Research Institute, Chinese Academy of Agricultural Sciences, Xinxiang, China; ^2^Department of Soil Science, Sindh Agriculture University, Tando Jam, Pakistan; ^3^Agricultural Clean Watershed Research Group, Institute of Environment and Sustainable Development in Agriculture, Chinese Academy of Agricultural Sciences, Beijing, China; ^4^Institute of Environment and Sustainable Development in Agriculture, Chinese Academy of Agricultural Sciences, Beijing, China; ^5^College of Plant Sciences, Tarim University, Alar, China; ^6^Forschungszentrum Jülich, Jülich, Germany; ^7^Department of Plant Breeding and Genetics, Sindh Agriculture University, Tando Jam, Pakistan

**Keywords:** acidic biochar, bacterial community, maize, rhizosphere, salt stress

## Abstract

Biochar has extensively been used for multiple purposes in agriculture, including improving soil microbial biomass. The current study aimed to investigate the effect of acidic biochar on maize seedlings’ rhizosphere bacterial abundance under salinity. There were seven treatments and three replicates in a controlled greenhouse coded as B0S1, B1S1, and B2S1 and B0S2, B1S2, and B2S2. CK is control (free of biochar and salt); B0, B1, and B2 are 0, 15, and 30 g biochar (kg soil)^–1^; and S1 and S2 are 2.5 and 5 g salt pot^–1^ that were amended, respectively. After harvesting the maize seedlings, the soil samples were collected and analyzed for soil microbial biomass, bacterial abundance, and diversity. The results revealed that relative abundance of Proteobacteria, Actinobacteria, and Chloroflexi increased on phylum level, whereas *Actinomarinales*, *Alphaproteobacteria*, and *Streptomyces* enhanced on genus level, respectively, in B2S1 and B2S2, when compared with CK and non-biochar amended soil under saline conditions. The relative abundance of *Actinomarinales* was positively correlated with total potassium (TK) and *Gematimonadetes* negatively correlated with total phosphorus (TP). Biochar addition slightly altered the Ace1, Chao1, and alpha diversity. Principal component analysis corresponded to the changes in soil bacterial community that were closely associated with biochar when compared with CK and salt-treated soils. In conclusion, acidic biochar showed an improved soil microbial community under salinity.

## Introduction

Soil salinization has become one of the serious environmental challenges that adversely affect crop growth and soil properties. Salinity can severely affect plant health and yield by causing an imbalance in nutrient uptake, by increasing the negative osmotic water pressure on plant cells (Yaish et al., 2016) and by reducing nutrient availability in the soil (Moradi et al., 2011). Salinity affects plants in two ways, i.e., osmotic pressure and ion imbalance (excessive uptake of Na^+^ or Cl^–^). It reduces not only Ca^2+^ availability but also Ca^2+^ transport and mobility from the roots to the other growing regions of plants, which decreases the quality of both plants’ vegetative and reproductive organs. Salinity can directly affect nutrient uptake, such as Na^+^ reducing K^+^ uptake or Cl^–^ reducing NO_3_^–^ uptake. It can also cause complex interactions that affect plant metabolism, susceptibility to injury or internal nutrient requirement (Grattan and Grieve, 1998; Munns and Tester, 2008), and decreases soil organic matter, exchangeable K^+^, and soil microbial biomass (Zhang et al., 2019). It has been observed by different studies that soil respiration, enzyme activity, bacterial growth, and nutrient cycling have been negatively influenced by salinity (Tripathi et al., 2006; Rousk et al., 2011; Yaish et al., 2016).

The processes that occur within the soil rhizosphere are closely related to soil microorganisms. Soil microorganisms, such as bacteria and fungi, control the ecosystem functioning through decomposition and nutrient cycling and may serve as indicators of land-use change and ecosystem health (Doran and Zeiss, 2000; Waldrop et al., 2000; Yao et al., 2000). Soil organisms are the most important environmental creatures that spend all or part of their lives in the soil and help to improve soil quality. They maintain the biological activity of the soil and guarantee soil nutrient cycling and soil structure formation (Qin et al., 2019). Each handful of soil contains billions of organisms, with representatives of nearly every phylum of microorganisms (Brady and Weil, 2002). Soil microorganisms make up less than 0.5% (w/w) of the soil mass, but they play a crucial role in soil properties and processes (Yan et al., 2015). They play a pivotal role in soils through the mineralization of organic matter into plant nutrients. However, several biotic and abiotic factors affect soil microorganisms, microbial properties, community structure, and functions (Szoboszlay et al., 2019; Kumar et al., 2020; Wang et al., 2021); salinity is one of them, which negatively influences them by soluble salts *via* two osmotic effect and specific ions (Na^+^ and Cl^–^) effects (Yan et al., 2015). It was observed by Wang et al. (2021) in their greenhouse experiment that the increasing level of salinity (15 and 30 mS cm^–1^) decreased the abundance of bacteria with denitrification function. In contrast, Wu et al. (2021) revealed that the combined application of organic and mineral fertilizers increased the microbial biomass carbon under saline–alkaline soil. Another study showed that 4 years’ consecutive application of biochar significantly enhanced microbial biomass carbon (Zhang et al., 2014).

Biochar is a multifunctional substance used in agriculture obtained by a process called pyrolysis from various organic originated materials (Lehmann and Joseph, 2015; Palansooriya et al., 2019). Biochar is a carbon-rich, fine-grained, porous substance, which basically comprises nanostructured aromatic compounds systematically arranged like graphite (Islam et al., 2018). Biochar has been a novel approach and used for various purposes. It is applied to enhance soil fertility, water uptake, bulk density, soil microbial community, and soil carbon sequestration and mitigate the greenhouse effect (Zhang et al., 2014; Akhtar et al., 2015; Palansooriya et al., 2019; Ibrahim et al., 2020). Biochar has been used to improve growth and grain yield (Sial et al., 2019; Ibrahim et al., 2020) in the salt-stressed environment. However, many field and greenhouse experimental studies have reported the mitigating effect of biochar on plant growth, soil properties, and microbial biomass under salinity stress (Yao et al., 2000; Lashari et al., 2013; Akhtar et al., 2015; Tan et al., 2019; Ibrahim et al., 2020). The previous studies having low pH biochar only focused on the physical and chemical properties of the soil (Esfandbod et al., 2017; Mierzwa-Hersztek et al., 2017).

To date, no information is available about the application of acidic pH biochar for soil microbial communities under salinity stress. Hence, the present experiment has been conducted in a phytotron to determine the impact of acidic biochar on salt-affected soil microbial community and bacterial abundance.

## Materials and Methods

### Soil and Salt Samplings

The surface soil was collected from an Apple Orchard of Alar City, Xinjiang, China. The soil was processed for physicochemical properties before the commencement of the experiment. The soil was analyzed for pH and electrical conductivity (EC) in 1:5 w/v extract by using pH and EC meter (Fisher Scientific, United States); soil texture was determined by hydrometer method (Bouyoucos, 1962), cation exchange capacity of the soil samples was measured by ammonium acetate method (Thomas, 1983), and total NPK was measured by an elemental analyzer. Salt was also collected from Alar City; the EC of the salt was 17 dS m^–1^ and analyzed for selected chemical properties, i.e., chloride, potassium, sodium, and magnesium, before using for the experiment (Table 1).

**TABLE 1:** Physicochemical properties of soil, wood biochar, and salt used in experiment.

Parameters	Soil	Biochar	Salt
Texture	Silty clay loam	–	–
pH	8.51	2.52	–
EC (dS m^–1^)	3.31	–	–
CEC cmol (+) kg^–1^	3.86	23.7	–
Total Nitrogen (mg g^–1^)	0.50	2.8	–
Total Phosphorus (mg g^–1^)	0.66	64.9	–
Total Potassium (mg g^–1^)	–	5.6	–
Chloride (g kg^–1^)	7.10	0.03	70.0
Exchangeable K^+^ (g kg^–1^)	–	–	0.02
Exchangeable Na^+^ (g kg^–1^)	18.13	0.50	4.46

### Collection of Biochar and Its Characterization

Acidic wood biochar was collected from Shangqiu SanLi Company, Henan Province of China, which was pyrolyzed at 250–300°C in an oxygen-free environment (Kiln). Biochar pH was determined in water extract (1:10 w/v) with a pH meter by the method proposed by Dai et al. (2013). Cation exchange capacity was measured by the ammonium acetate method (Thomas, 1983), and total NPK and Na^+^ were determined by the acid digestion method (Hongyan et al., 2015); an extract of the samples was run on Continuous Flow-Analyzer AA3. The characteristics of biochar are given in Table 1.

### Experimental Site and Treatments

The experiment was conducted in a controlled greenhouse during 2019–2020 at the Experimental Station of Farmland Irrigation Research Institute, Chinese Academy of Agricultural Sciences, Qiliying, Xinxiang, China. The greenhouse was maintained at 25/20°C for day/night temperature with a photoperiod of 14 h. The PVC pots of 15-cm width and 25-cm height were filled with a 5-kg mixture of soil and biochar. The experimental treatments comprising two biochars and two salt levels were arranged in a completely randomized design with a 2 × 2-factorial scheme. In this study, seven treatments were arranged with three replications, including control (CK without biochar and salt), and biochar was added 0, 15, and 30 g kg^–1^ soil followed by the application of salt solution prepared with 2.5 and 5 g pot^–1^ in 1 L of water; the overall treatments were termed as CK, B0S1, B1S1, B2S1, B0S2, B1S2, and B2S2. The pots were irrigated before sowing and left for field capacity. Three seeds of maize variety (cv. Denghai605) were sown in each pot. After successful germination, one plant was maintained per pot. Fertilization was done with Hoagland solution on the 7th and 15th day after germination.

### Soil Sampling and Microbial Measurement After Harvest

Thirty (30) days after sowing, agronomical, physiological, and chemical parameters were collected, and the plant was harvested. For the microbial community, soil samples were collected at a depth of 10 cm using a stainless soil auger (3.5 cm) around the rhizosphere from each pot in tight plastic tubes and were kept in liquid nitrogen to minimize the risk of decaying activity of bacterial species. Such a scheme of sampling and preservation for microbial analysis has also been adopted previously (Yin et al., 2021; Zhang et al., 2021; Zhao et al., 2021). The collected samples were immediately transported to the laboratory and stored at −80°C for soil DNA extraction. In brief, microbial community genomic DNA was extracted from 0.5-g soil using the E.Z.N.A.^®^ soil DNA Kit (Omega Bio-tek, Norcross, GA, United States) according to manufacturer’s instructions. The DNA extract was checked on 1% agarose gel, and DNA concentration and purity were determined with NanoDrop 2000 ultraviolet–visible spectrophotometer (Thermo Scientific, Wilmington, NC, United States). The hypervariable region V3-V4 of the bacterial 16S ribosomal RNA (rRNA) gene was amplified with primer pairs 338F (5′-ACTCCTACGGGAGGCAGCAG-3′) and 806R (5′GGACTACHVGGGTWTCTAAT-3′) by an ABI GeneAmp^®^ 9700 polymerase chain reaction (PCR) thermocycler (ABI, CA, United States). The PCR amplification of the 16S rRNA gene was performed as follows: initial denaturation at 95°C for 3 min, followed by 27 cycles of denaturing at 95°C for 30 s, annealing at 55°C for 30 s and extension at 72°C for 45 s, and single extension at 72°C for 10 min, and end at 4°C. The PCR mixtures contain 5 × *TransStart* FastPfu buffer 4 μl, 2.5-mM deoxynucleoside triphosphates 2 μl, forward primer (5 μM) 0.8 μl, reverse primer (5 μM) 0.8 μl, *TransStart* FastPfu DNA Polymerase 0.4 μl, template DNA 10 ng, and finally double-distilled water up to 20 μl. PCR reactions were performed in triplicate. The PCR product was extracted from 2% agarose gel and purified using the AxyPrep DNA Gel Extraction Kit (Axygen Biosciences, Union City, CA, United States) according to manufacturer’s instructions and quantified using Quantus™ Fluorometer (Promega, United States).

### Illumina Miseq Sequencing

Purified amplicons were pooled in equimolar and paired-end sequenced on an Illumina MiSeq PE300 platform/NovaSeq PE250 platform (Illumina, San Diego, CA, United States) according to the standard protocols by Majorbio Bio-Pharm Technology Co., Ltd., (Shanghai, China).

### Processing of Sequencing Data

The raw 16S rRNA gene sequencing reads were demultiplexed, quality-filtered by fast version 0.20.0 (Chen et al., 2018), and merged by FLASH version 1.2.7 (Magoč and Salzberg, 2011) with the following criteria: (i) the 300-bp reads were truncated at any site receiving an average quality score of < 20 over a 50-bp sliding window, and the truncated reads shorter than 50 bp were discarded; reads containing ambiguous characters were also discarded; (ii) only overlapping sequences longer than 10 bp were assembled according to their overlapped sequence. The maximum mismatch ratio of the overlap region is 0.2. Reads that could not be assembled were discarded; (iii) samples were distinguished according to the barcode and primers, and the sequence direction was adjusted, exact barcode matching, two nucleotide mismatches in primer matching.

Operational taxonomic units (OTUs) were clustered with 97% similarity cutoff (Stackebrandt and Goebel, 1994; Edgar, 2013) using UPARSE version 7.1 (Edgar, 2013), and chimeric sequences were identified. The taxonomy of each OTU representative sequence was analyzed by RDP Classifier version 2.2 (Wang et al., 2007) against the 16S rRNA database (e.g., Silva v138) using a confidence threshold of 0.7.

### Statistical Analysis

The data were subjected to one-way analysis of variance to SPSS version 23.0 (IBM Corporation, NY, United States) and presented as the mean values of three replicates and standard error using Tukey’s test. There were significant differences in soil properties, alpha diversity, and bacterial relative abundances among treatments. Pearson’s correlation was used to determine the relationships between nutrients and bacterial phylum. OTUs were analyzed for alpha and beta diversity for bacteria. Principal component analysis (PCA) on the relative abundances of biochar and salt stress was used to assess differences in the microbial community structure among all the treatments.

## Results

### Effect of Biochar on Microbial Community on Phylum Level

**FIGURE 1:**
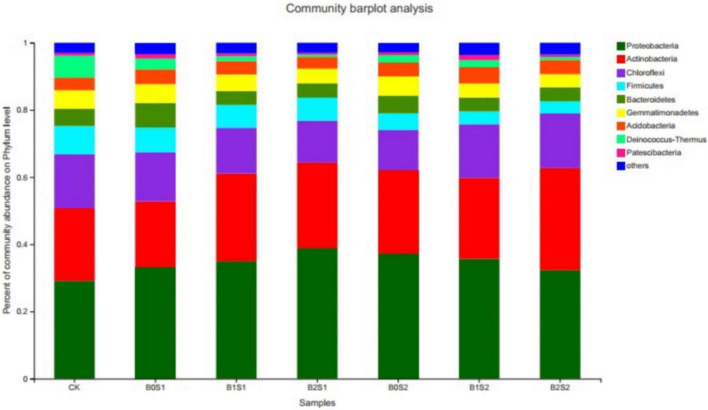
Relative abundance of soil bacterial communities at phylum level. Data are presented mean; CK = control, B0S1, B1S1, B2S1, B0S2, B10S2, and B2S2 = 0, 15, and 30 g biochar + 0.5 and 1 g salt g kg^–1^, respectively.

The relative abundance was Proteobacteria (34.6%), Actinobacteria (24.7%), Chloroflexi (14.4%), Firmicutes (6.0%), Gemmatimonadetes (4.9%), Bacteroidetes (4.9%), and Acidobacteria (4.0%), which represented approximately 94% of the total relative abundance (Figure 1). The Proteobacteria, Actinobacteria, and Chloroflexi emerged as having the highest number of bacterial communities among all the bacterial phyla, which accounted for 73.6% of the total bacterial relative abundance. The highest number of Proteobacteria (39.0%) was recorded in the soil amended with biochar (B2S1). The application of biochar increased the relative abundance of Proteobacteria and Actinobacteria when compared with CK. Biochar amendment at a low salt level (0.5%) also enhanced the relative abundance of Proteobacteria. In contrast, the same biochar treatments (15 and 30 g kg^–1^ biochar) decreased the relative abundance of Proteobacteria at high salt levels (1%). However, Actinobacteria showed an increasing trend at both biochar levels when compared with non-biochar-treated soil samples. However, the rest of the bacterial community in all phyla decreased as compared with CK and biochar-treated soils, whereas it improved in the saline environment. Both biochar levels with both salt levels (2.5 and 5) decreased biomass of the Firmicutes, Gemmatimonadetes, Bacteroidetes, Acidobacteria, Deinococcus-Thermus, and Patescibacteria.

### Effect of Biochar on Microbial Community on Genus Level

**FIGURE 2:**
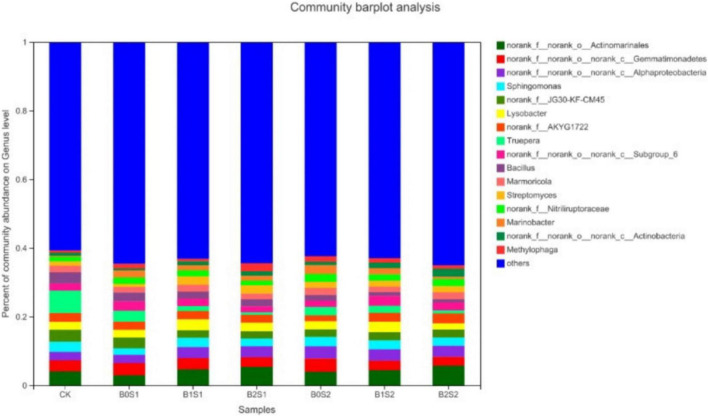
Relative abundance of soil bacterial communities at genus level. Data are presented mean; CK = control, B0S1, B1S1, B2S1, B0S2, B10S2, and B2S2 = 0, 15, and 30 g biochar + 0.5 and 1 g salt g kg^–1^ respectively.

Seventeen dominant genera were found in all treatments (Figure 2). The relative abundance of *Actinomarinales*, *Gemmatimonadetes*, *Alphaproteobacteria*, *Sphingomonas*, and *JG30-KF-CM45* remained dominant, but only *Actinomarinales*, *Alphaproteobacteria*, and *Streptomyces* were higher in biochar-treated soils compared with non-biochar-treated soils and CK, respectively. The relative abundance of *Actinomarinales*, *Alphaproteobacteria*, and *Streptomyces* under B0S1, B1S1, and B2S1 and B0S2, B1S2, and B2S2 showed 2.97, 4.74, and 5.37%; 3.98, 4.44, and 5.74%; 0.86, 2.48, and 2.43%; and 1.80, 170, and 1.86%, respectively. In contrast, most of the relative abundance of genera significantly declined in biochar-treated soils when compared with salt-treated soils and CK.

**FIGURE 3:**
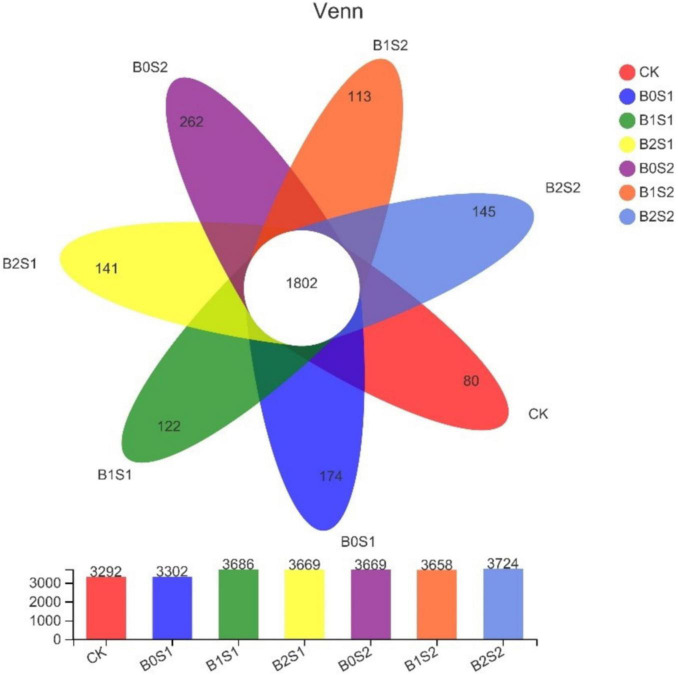
Venn diagram analysis of abundant and showing shared operational taxonomic units. Data are presented mean; CK = control, B0S1, B1S1, B2S1, B0S2, B10S2, and B2S2 = 0, 15, and 30 g biochar + 0.5 and 1 g salt g kg^–1^ respectively.

After sequencing quality control, a total of 2,839 OTUs were identified from all soil samples. Unique OTUs were found in salt-treated soils (Figure 3). The figure showed that biochar application improved the OTUs in soil samples when compared with CK. In contrast, high OTUs were recorded in B0S1 (262) and B0S2 (174), where the soil was amended with salt levels. The soil amended with biochar (B1S2) showed the lowest number of OTUs.

### Effect of Biochar on Soil Bacterial Community Richness and Diversity

The one-way analysis of variance showed no significant difference between Ace1 and Chao1 richness and Good’s coverage percentage among all the treatments. A slight change was observed for Shannon and Simpson diversity among all the treatments (Table 2). The highest value for Ace1 was recorded in the biochar-treated soil under B2S2 (3,860.19), and the lowest was noticed in CK (3,171.44). Similar results can be seen under the same treatments for Chao1 (Table 2). Statistically, a significant change was observed in B0S1 (6.31) and CK (0.008) for Shannon and Simpson diversity, respectively, when compared with other biochar- and non-biochar-treated soils. The Good’s coverage percentage was the same in all treatments, including CK. The Shannon index in biochar-treated pots was significantly higher than in the CK, whereas the Shannon index was greater in B0S2, B1S2, and B2S2 with increasing biochar levels. Acidic biochar addition decreased Shannon index.

**TABLE 2:** Effect of acidic biochar on Ace and Chao1 richness and Shannon and Simpson diversity and Good’s coverage (%).

Treatments	Richness		Alpha Diversity		Coverage (%)
	Ace 1	Chao1	Shannon	Simpson	
CK	3,171.44	3,203.57	6.08	0.008	0.98
B0S1	3,441.23	3,389.60	6.31	0.005	0.98
B1S1	3,652.72	3,494.83	6.21	0.005	0.98
B2S1	3,712.12	3,539.77	6.21	0.005	0.98
B0S2	3,251.28	3,104.12	6.14	0.006	0.98
B1S2	3,860.19	3,551.11	6.20	0.006	0.98
B2S2	3,738.81	3,539.33	6.25	0.005	0.98

### Relationship Between Soil Microbial Structure and Acidic Biochar

**FIGURE 4:**
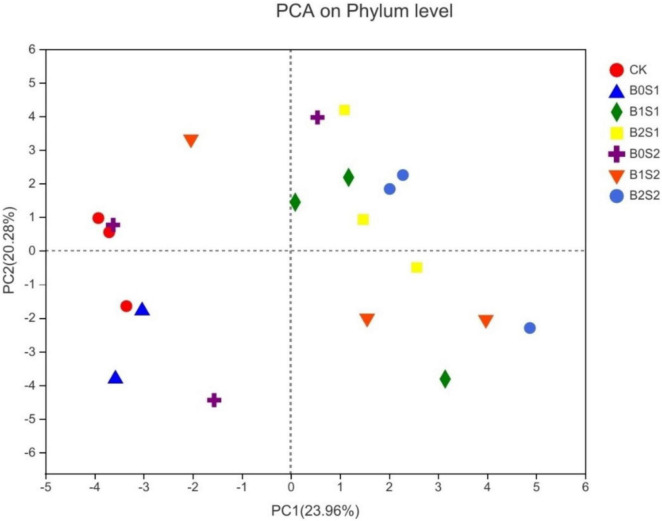
Principal component analysis for microbial communities of soil amended with biochar and non-biochar. Data are presented mean; CK = control, B0S1, B1S1, B2S1, B0S2, B10S2, and B2S2 = 0, 15, and 30 g biochar + 0.5 and 1 g salt g kg^–1^ respectively.

Principal component analysis was used to elucidate the relationship between soil microbial structure and biochar. The PCA showed distinct differences among treatments (Figure 4). The PCA1 (23.96%) and PCA2 (20.28%) jointly explained 44.24% of the total community variability. Figure 4 showed a good variation for microbial community structure among all the treatments. B2S1 and B2S2 had more separation when compared with CK, whereas B2S1 had also shown a similar trend compared with B0S1 and B0S2, respectively.

### Relationship Between Acidic Biochar and Bacterial Genera

**FIGURE 5:**
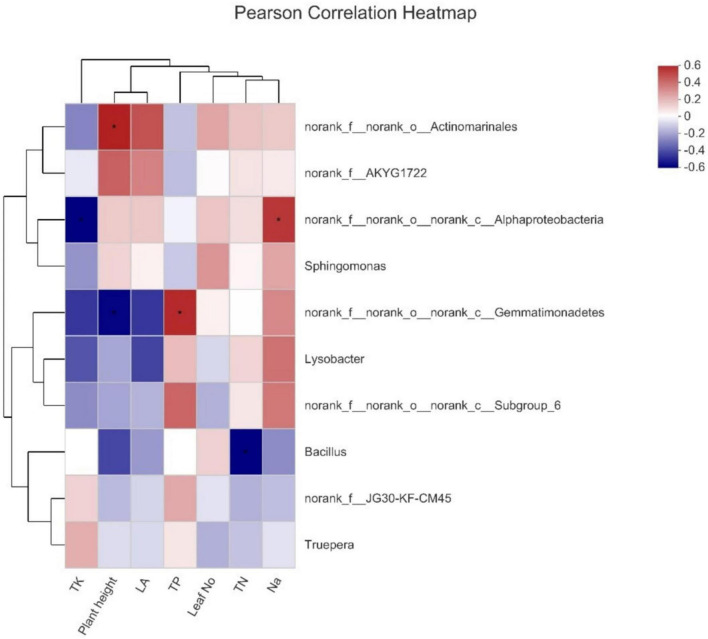
Pearson correlation coefficients among nutrients and bacteria abundance.

The Pearson test was conducted to study the connection between environmental factors and dominant genera (Figure 5). It is observed that *Actinomarnales* was the primary genera and had a significant positive correlation with plant height, and Alphaproteobacteria was found to have a positive and negative correlation with Na^+^ and total potassium (TK), respectively, whereas Gemmatimonadetes also had a positive correlation with total phosphorus (TP); however, a negative correlation was observed in case of plant height. *Bacillus* showed a negative correlation with total nitrogen (TN).

## Discussion

### Effect of Biochar on Microbial Community on Phylum and Genus Levels

Soil microbial biomass is considered a labile soil organic fraction and an important source and sink for plant-available nutrients (Ajwa et al., 1999). The survival of microorganisms is important for plant growth, yield, and soil properties, and most of the microbes spend their life cycle in the rhizosphere because it is the hot spot for microbial activity; carbon is available from plant roots exudates as well (Bais et al., 2006; Kuzyakov and Xu, 2013; Brtnicky et al., 2019). Not many studies on the effects of acidic biochar application on the soil microbial community have been reported. The experiment was conducted to evaluate the effect of acidic biochar on the rhizosphere microbial community under salinity stress. The present study revealed that acidic biochar has positively affected the soil microbial community in the rhizosphere. Proteobacteria and Actinobacteria on phylum level and Actinomarinales on genus level, respectively, were high with acidic pH of biochar (Figures 1, 2). The results are in accordance with Cho et al. (2016), who reported that Proteobacteria, Firmicutes, Acidobacteria, and Bacteroidetes were found to be the most abundant under acidic pH (4.1 and 5.3). In contrast, salinity had a stronger influence on the microbial community than biochar. This is because a high abundance of Firmicutes, Bacteroidetes, Gemmatimonadetes, Acidobacteria, Deinocococcus-Thermus, and Patescibacteria were found at high salinity. Some bacterial species may be salt dependent and/or salt tolerant (Yang et al., 2021). Therefore, some of the abundances of some bacterial species increased at high salt levels. The high salinity, low biochar rate, and application of biochar for a short duration (30 days only) to soil might be the reason for the low abundance of bacterial species. Some species of bacteria may take time to break down the organic C for the survival of microbiota. Different bacterial species show a different level of tolerance to salt even under the same phylum (Yang et al., 2021). The response of the microbial community to biochar application is dependent on its type, duration, soil conditions, and properties, such as biochar pH and pyrolysis temperature (Cho et al., 2016; Fan et al., 2020; Xu et al., 2021). Zhang et al. (2021) has reported that increasing some phylum (Bacteroidetes, Gemmatimondetes, and Firmicutes) and bacterial communities showed tolerance and strongly structured with increasing salinity stress. In contrast, Yang et al. (2021) revealed that extreme salinity levels (34.41 dS m^–1^) significantly decreased the abundance of Proteobacteria, Actinobacteria, and Acidobacteria. The pH has a great impact on the relative abundance of bacteria on phylum and genus levels (Wang et al., 2019). The pH was the main driving force for the structural diversity and abundance of soil microbial communities (Wang et al., 2019). Biochar application makes a favorable environment in the rhizosphere for microorganisms and the community structure of soil bacteria because of its porous nature (Warnock et al., 2010; Gao et al., 2017).

### Effect of Biochar on Bacterial Diversity and Richness Index

A number of factors affect the bacterial structure, such as a change in soil pH and salinity (Shi et al., 2019; Szoboszlay et al., 2019). The present experiment demonstrated that salinity negatively affected the bacterial community structure (Chao1, Ace1, and Shannon) (Table 1). However, biochar amendment increased the bacterial richness and diversity under salinity stress. The results of our study are in accordance with the findings of Yang et al. (2021), who reported that extreme salinity (34.41 dS m^–1^) lowered significantly Shannon, Ace, and Chao 1. However, Han et al. (2017) investigated that biochar application had shown a positive effect on the bacterial structure under saline conditions. The increase in bacterial diversity and richness might be due to labile C input, biochar surface properties, and change in pH reported by Nguyen et al. (2018) and Szoboszlay et al. (2019). Nguyen et al. (2018) indicated in their field study that bacterial diversity was increased with biochar amendment. The results of the current study are further supported by the results of Fan et al. (2020), who applied biochar and observed that biochar applied at 8% w/w enhanced Ace and Chao richness. When compared with CK and without biochar soil, it showed that Shannon and Simpson’s diversity was negatively affected by biochar. Rutigliano et al. (2014) showed that wood-derived biochar had no detrimental effect on microbial biomass, activity, and diversity because the addition of biochar for a short period could not affect soil microbial diversity.

### Relationship Between Biochar and Soil Microbial Community Structure

The relationship between soil microbial community and biochar has been reported in many studies (Han et al., 2017; Yu et al., 2018; Li et al., 2019; Fan et al., 2020; Wang et al., 2021). The present study shows that the relationship between biochar and microbial community structure was distinct under saline conditions. The reason for the distinct separation of community structures between treatments and control (biochar- and non-biochar-amended soil) might be due to high salinity. Liao et al. (2016) revealed that biochar treatments were different and separated when compared with control due to a higher rate of biochar (4.5 t ha^–1^). The results agree with the results of Luo et al. (2020), who revealed that biochar affected microbial community structure. Similarly, Luo et al. (2017) showed that higher biochar treatment increased phospholipid fatty acid profile score. In contrast, Elzobair et al. (2016) reported that hardwood biochar, rate, and fast pyrolysis to Aridisol did not affect microbial community, structure, and activities; it may vary soil type and application rate.

## Conclusion

In this research, the amendment of acidic biochar to the soil under salinity showed that biochar slightly improved the microbial community at phylum and genus levels. Acidic biochar enhanced the relative abundance of Proteobacteria, Actinobacteria, and Chloroflexi at the phylum level; Actinomarinales, Alphaproteobacteria, and Streptomyces increased at genus level under salt stress. Acidic biochar had not had a significant effect on bacterial community structure and diversity. The variations in bacterial communities were closely related to the amendment of biochar to soil TK and TP. Overall, the short-term biochar application has shown some positive effects and altered the soil bacterial community composition. Furthermore, the long-term impact of acidic biochar on soil microbial community and structure should be studied.

## Data Availability Statement

The datasets presented in this study can be found in online repositories. The names of the repository/repositories and accession number(s) can be found in NCBI SRP343350.

## Author Contributions

MuS: writing—original draft preparation and investigation. MuS and AH: methodology, investigation, formal analysis, software, validation, visualization, data curation, and writing—review and editing. YG and JS: conceptualization and design. AH, MFS, MaS, YF, RR, JKS, SB, SA, YG, and JS: writing—review and editing, conceptualization, and software. All authors contributed to the article and approved the submitted version.

## Conflict of Interest

The authors declare that the research was conducted in the absence of any commercial or financial relationships that could be construed as a potential conflict of interest.

## Publisher’s Note

All claims expressed in this article are solely those of the authors and do not necessarily represent those of their affiliated organizations, or those of the publisher, the editors and the reviewers. Any product that may be evaluated in this article, or claim that may be made by its manufacturer, is not guaranteed or endorsed by the publisher.
